# An alternative structured weight management protocol to rapid weight loss in mixed martial arts: a prospective interventional study of pre-competition weight management strategies in professional athletes

**DOI:** 10.3389/fnut.2025.1581698

**Published:** 2025-10-08

**Authors:** Clóvis Albuquerque Maurício, Guilherme Giannini Artioli, Aleksandro Ferreira Gonçalves, Rafael Pereira Azevedo Teixeira, Victor Vieira, Ciro José Brito, Rodrigo Cunha de Mello Pedreiro, Diego Ignacio Valenzuela Pérez, Esteban Ariel Aedo-Muñoz, Emanuela Pierantozzi, Bianca Miarka

**Affiliations:** ^1^Department of Fights, Federal University of Rio de Janeiro, Rio de Janeiro, Brazil; ^2^LANEB - Laboratory of Nutritional & Exercise Biology, Institute of Biomedical Sciences, University of São Paulo, São Paulo, Brazil; ^3^Department of Physical Education, Juiz de Fora Federal University, Juiz de Fora, Brazil; ^4^Department of Physical Education, Estácio de Sá University, Rio de Janeiro, Brazil; ^5^Facultad de Ciencias, Escuela de Kinesiología, Universidad Santo Tomás, Santiago, Chile; ^6^Metropolitan University of Educational Sciences, Ñuñoa, Chile; ^7^Department of Neurosciences, Rehabilitation, Ophthalmology, Genetics, and Maternal and Child Health, Università degli Studi di Genova, Genoa, Italy

**Keywords:** athletic performance, dehydration, dietary supplements, energy intake, fluid therapy, nutritional status, rehydration, sports nutritional sciences

## Abstract

In Mixed Martial Arts (MMA), a combination of combat techniques and physical demands takes center stage. Before competition, many athletes engage in rapid weight loss (RWL) strategies to qualify for lower weight classes, aiming to gain a competitive edge by facing smaller or less physically dominant opponents. This practice is driven by the belief that regaining weight after the official weigh-in enhances strength, power, and resilience during the bout. For MMA athletes, adhering to an evidence-based Weight Management Protocol (WMP), targeting a 10% body weight reduction within 7 days prior to weigh-in, poses a significant challenge. This study evaluated the efficacy and safety of a structured nutritional strategy, including controlled dietary interventions and fluid manipulation, tailored for athletes seeking to lose 10% of body mass, along with evidence-based recommendations for post-weigh-in recovery. We followed 31 professional MMA athletes (28 males, aged 28 ± 4 years), all with established experience in RWL protocols. The study was divided into two phases: pre- and post-RWL assessments. Athletes achieved a mean weight reduction of 7.25 kg (10.6%) within the 7 days leading up to the weigh-in, conducted 8 days before competition. They demonstrated a mean weight regain of 7.5 kg (11.2%) within 30 h post-weigh-in, indicating effective rehydration and recovery. Starting 7 days before the weigh-in, athletes consumed 7 liters of fluids per day, with intake gradually decreasing. On the day before the weigh-in, they consumed only 0.5 liters. Following adherence to the protocol, 67.7% (21 athletes) secured victory in their bouts. This study presents a structured, athlete-centered nutritional approach for RWL, offering a potentially safer and more effective alternative to uncontrolled practices. Future research should explore the physiological, psychological, and performance-related implications of such methods through larger, randomized controlled trials.

## 1 Introduction

Mixed Martial Arts (MMA) is a modern combat sport that integrates various disciplines, such as boxing, Brazilian jiu-jitsu (BJJ), judo, Muay Thai, and wrestling [Ultimate Fighting Championship [UFC] (1)]. Since the inaugural UFC event in the United States in 1993, MMA has rapidly gained worldwide popularity, with leading professional organizations broadcasting events to over 165 countries in 40 languages (1). Due to its unique rule set and the combination of diverse combat styles, MMA bouts can unfold across multiple domains, including standing combat, clinching, ground fighting, and grappling (2). The primary objective is to knock out or submit opponents using striking and grappling techniques, both standing and on the ground (3).

Like other combat sports such as wrestling, BJJ, judo, and boxing, MMA features weight classes to promote fair competition and mitigate injury risks stemming from disparities in weight, strength, and body mass. This system ensures that greater body mass does not confer an undue advantage (4, 5). Many professional MMA events, adhering to standard UFC regulations, feature 12 weight divisions for both men and women. Athletes are allowed a single official weigh-in, during which they must meet their designated weight class limits (1).

Research indicates that most MMA athletes intentionally lose significant amounts of weight before weigh-ins, a practice known as rapid weight loss (RWL), typically occurring within 7 days (6, 7). Prior studies across various combat sports report that athletes commonly lose around 5% of their initial body weight through RWL (8, 9). Popular RWL methods among MMA athletes and other combat sport competitors include food restriction, fasting, and dehydration techniques, such as sauna use, hot baths, water loading, and fluid restriction (6, 10, 11). However, these strategies can lead to adverse effects, including cramps, fatigue, palpitations, fainting, dizziness, stomachaches, and headaches (12).

The rationale behind RWL is multifaceted. Some athletes employ RWL to gain a perceived size and strength advantage over opponents (6, 7), while others view it as a means to maintain fairness in a sport where RWL is widespread (13). Despite its potential harms, many athletes believe that RWL boosts confidence and enhances focus, determination, and aggression during competition, suggesting possible psychological benefits (14, 15).

To mitigate the health risks associated with RWL, some researchers advocate for gradual weight loss over several weeks before competition (16). Studies suggest that a 30% reduction in energy intake, paired with protein consumption of ≥1.2 g/kg/day, can promote fat loss while preserving lean mass (17). The most common nutritional strategies for RWL include reducing energy, carbohydrate, and fat intake, limiting fiber, maintaining adequate protein levels, and restricting sodium intake. These, combined with active dehydration techniques, aim to achieve approximately 10% body weight reduction within 7 days (18, 19). Proper macronutrient adjustment and individualized caloric plans are crucial, underscoring the role of sports nutritionists in guiding the process. However, most of these approaches are based on surveys or short-term protocols that do not integrate structured nutritional planning with hydration control under professional supervision (18, 19). Most of the studies have not verified protocols applied in real-world competitive settings with continuous follow-up. This gap limits the development of standardized interventions capable of reducing the health risks associated with RWL.

Weight losses in MMA often exceed those observed in other combat sports, such as boxing, BJJ, and judo, sometimes surpassing 10% of initial body weight (19, 20). This underscores the need to understand the potential health risks and performance impairments associated with RWL in MMA athletes.

Recent reports in the media and scientific literature have documented severe health events, including hospitalizations and even athlete fatalities, associated with poorly managed RWL, reinforcing the urgency of establishing safer, standardized alternatives. For instance, Lakićević et al. (21) found that losing 5% of body mass within 1 week can impair kidney function, evidenced by elevated creatinine and blood urea nitrogen levels. Furthermore, energy restriction during RWL may compromise bone health. Prouteau et al. (22) observed that judokas experiencing a 4% body mass loss exhibited increased circulating glucocorticoids and bone resorption markers, potentially compromising bone integrity long term. Regarding psychological factors, RWL has been linked to increased perceived anger and fatigue (6, 23). Moreover, Kim and Park (18) found an association between RWL and an elevated risk of sport-related injuries among wrestlers. Thus, RWL should be conducted under professional supervision and generally discouraged (13).

We hypothesize that the implementation of a structured and professionally supervised Weight Management Protocol (WMP) will enable MMA athletes to achieve effective rapid weight loss (RWL) and full recovery within 30 h post-weigh-in, while minimizing risks to health and performance. When applied in real-world competitive settings, this protocol is expected to offer new insights into safer and more practical alternatives to traditional, unregulated RWL methods.

Therefore, the present study aimed to contribute a novel, structured WMP and to evaluate nutritional strategies designed to facilitate a 10% body mass reduction. This protocol integrates controlled dietary interventions, hydration strategies, and post-weigh-in recovery plans tailored to the specific needs of athletes.

## 2 Materials and methods

### 2.1 Study design

The present study is a prospective interventional case study focusing on professional MMA athletes. Recruitment began with an initial assessment that included nutritional anamnesis, anthropometric evaluation, and training volume analysis. The Weight Management Protocol (WMP) was adjusted by Wright and Garthe (24), and was divided into three distinct phases: pre-rapid weight loss (RWL), RWL, and post-RWL recovery. Each phase was defined by specific dietary, hydration, and training targets, ensuring a gradual transition to minimize physiological stress.

The pre-RWL phase commenced 6 weeks before competition, involving body weight monitoring alongside personalized dietary and hydration planning. Protein intake was adjusted, emphasizing a balanced macronutrient composition of carbohydrates, proteins, and fats. Hydration was regulated throughout the day, with increased intake during training sessions.

**Figure 1:**
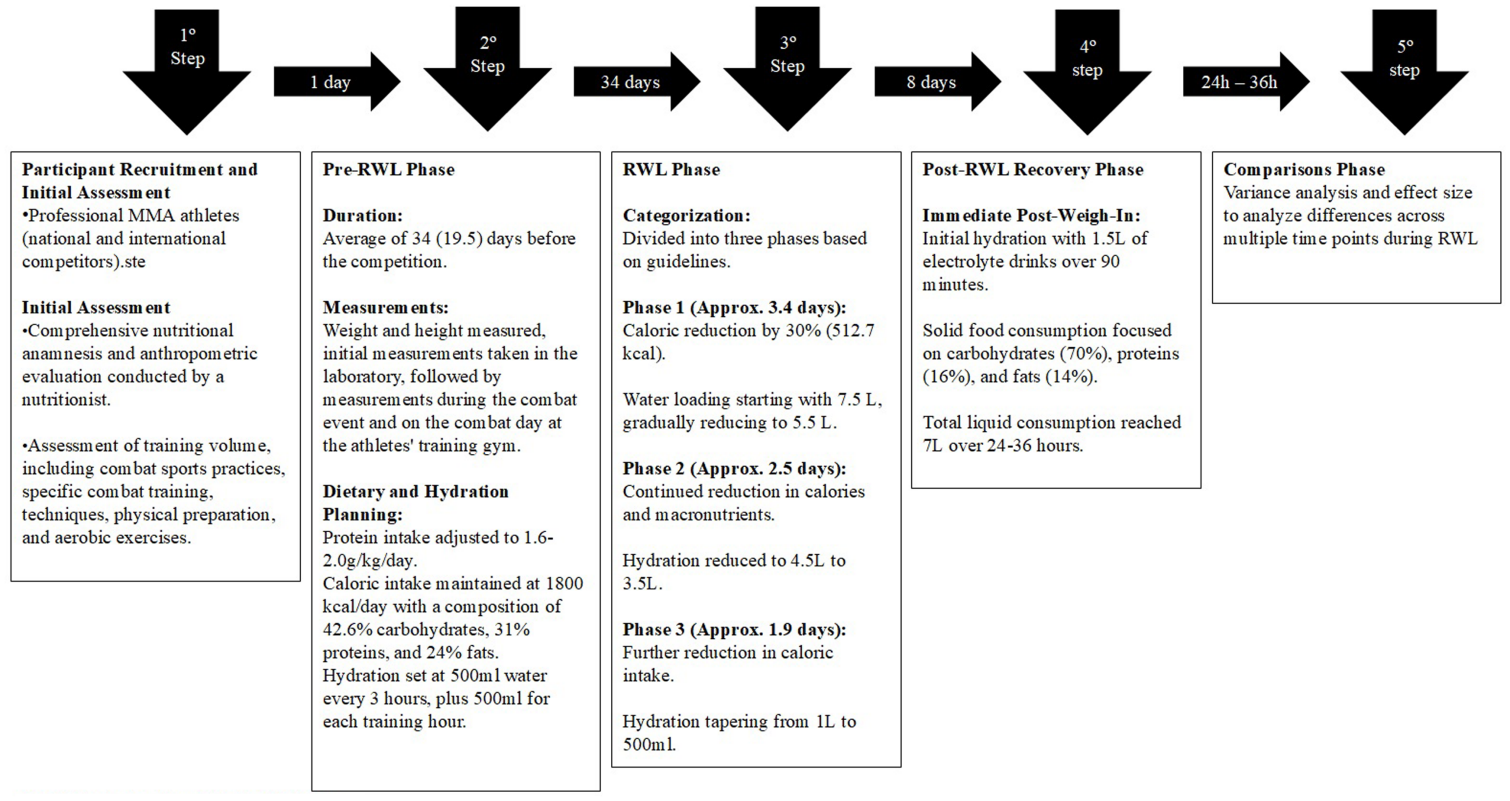
Study design of Weight Management Protocol (WMP) in professional MMA athletes. MMA mixed martial arts, RWL Rapid Weight Loss.

The RWL phase was further subdivided into three progressive stages: The first stage involved significant energy restriction alongside an initial phase of water loading. The second stage continued with further reductions in both energy and fluid intake, while the final stage involved a gradual tapering of hydration. The post-RWL recovery phase prioritized rehydration with electrolyte drinks, carbohydrate-rich foods, and structured liquid intake throughout the recovery period, everyone made the same hydration strategy, even on the pre weight-in and after weight-in Rationale for electrolyte composition followed Sawka et al. (25) guidelines for optimal post-exercise rehydration. Everyone received text messages every week and on the weight day about the diet and the results, like a healthy feeling and diet adherence. The study concluded with an analysis of variance (ANOVA) and effect size calculations to assess changes throughout the RWL process. To verify potential confounding variables, a General Linear Model (GLM) was used to examine the effects of age and sex on body mass across the RWL phases, training volume, and prior weight loss experience. Figure 1 illustrates the study design.

### 2.2 Participants

Thirty-one Brazilian professional MMA athletes (28 males and 3 females), with an average age of 28 ± 4 years, participated in this case study. The majority (77.4%) were preparing for national events, while 22.6% trained for international competitions. All athletes were from Rio de Janeiro and sought nutritional guidance for effective weight management and recovery for their upcoming professional MMA bouts. Data collection occurred between April 2021 and April 2023, spanning two phases: an average of 34 (±19.5) days before and immediately after RWL. Although this was a convenience sample, the sample size is consistent with similar interventional studies involving professional MMA athletes (20, 26), which typically include 15–30 participants. Given the controlled context of the intervention and real-world application, the number was considered appropriate to explore the protocol’s feasibility and effectiveness.

Table 1 presents the athletes’ training volume, encompassing all combat sports practices, specific technical training, physical preparation, and aerobic conditioning.

**Table 1:** Characterization of the MMA participants.

Characterization	
Participants (W)	28 (3)
Age (Years)	27.8 (3.8)
Height (m)	1.76 (0.08)
Professional experience	
National	24 (77.4%)
International	7 (22.6%)
Times you have done RWL	9.2 (6.2)
How long have you been practicing MMA? (years)	
1 < 3	4 (12.9%)
3 < 5	11 (35.5%)
5 < 10	9 (29%)
10<	7 (22.5%)
How many hours a week do you train?	
4,5 < 6 h	7 (22.5%)
6 < 9 h	2 (6.5%)
9 h < 12 h	14 (45%)
>12 h	8 (25.8%)
Result	21 W; 8 L; 2 D

To be eligible for inclusion, participants had to be adult MMA competitors with at least 1 year of professional experience, prior RWL experience for MMA competitions, and an upcoming scheduled bout requiring weight reduction. Screening included medical history evaluations and interviews to confirm eligibility. Exclusion criteria included a history or current use of medications or anabolic steroids, as well as injuries preventing competition. All participants were informed about study procedures, potential risks, and benefits before providing written informed consent. Participants were not financially compensated but received personalized nutritional guidance throughout the study. The study received approval from the local Research Ethics Committee and adhered to national legislation and the Declaration of Helsinki (no. 57395822.0.0000.5286).

### 2.3 Measurements

Weight measurements were conducted using a calibrated digital scale (Multilaser®, São Paulo, SP), while height was self-reported. The scale used was certified for clinical use and recalibrated weekly. Previous studies in sports science literature have used similar or identical equipment, and this method has shown high reliability for short-term tracking of body mass fluctuations in combat athletes (16). Initial weight measurements were taken in the laboratory, with subsequent measurements taken during the combat event and on the day of competition at the athletes’ training facility. Hydration was assessed and managed through a fractional water consumption strategy, with standardized volumes (500 mL every 3 h, plus 500 mL per training hour), based on validated guidelines from Sawka et al. (25) for rehydration in high-performance sports contexts.

#### 2.3.1 Nutrition and hydration monitoring

All nutritional data were collected and analyzed by a certified sports nutritionist using validated tools. The TBCA® (27) served as the primary database for dietary intake calculations, which has been widely accepted in Brazilian sports nutrition research.

Nutritional Anamnesis and Assessment: During the initial stage, athletes underwent a comprehensive nutritional review, including competitive experience, habitual dietary patterns, and an anthropometric assessment conducted by a certified nutritionist.

Dietary Planning: Following the assessment, individualized diet plans were developed for each athlete, considering their habitual eating patterns, financial resources, and training schedules. Dietary adjustments were tailored by a nutritionist, focusing on protein intake aligned with the recommended 1.6 to 2.0 g/kg/day (28). Energy intake was maintained based on the initial dietary recall, with macronutrient distribution comprising 42.6% carbohydrates, 31% protein, and 24% fat. Before the RWL period, the average energy intake was set at 1,800 kcal/day without an energy deficit, as detailed in Table 2.

**Table 2:** Distribution RWL phases, with caloric and macronutrient.

Initial diet	Mean	SD	Percentages
Days	29.2	17	
Cal [Kcal]	1812.3	501.7	
Ptn [gm]	141.9	33.2	31.3
Cho [gm]	194.1	84.2	42.8
Lpd [gm]	46.9	17.1	23.3
Fiber [gm]	30.3	12.4	
1st RWL PHASE			
Days	3.5	1.0	
Cal [Kcal]	1299.7	305.4	
Ptn [gm]	125.3	33.6	38.6
Cho [gm]	112.5	44.2	34.6
Lpd [gm]	32.8	9.4	22.7
Fiber [gm]	18.8	8.4	
2nd RWL PHASE			
Days	2.5	0.5	
Cal [Kcal]	981.3	154.7	
Ptn [gm]	111.2	21.6	45.3
Cho [gm]	55.0	17.9	22.4
Lpd [gm]	30.4	9.2	27.8
Fiber [gm]	10.0	3.4	
3rd RWL PHASE			
Days	2.0	0.3	
Cal [Kcal]	669.5	167.6	
Ptn [gm]	86.4	21.2	51.6
Cho [gm]	29.1	12.0	17.4
Lpd [gm]	22.6	7.0	30.4
Fiber [gm]	5.2	2.8	

#### 2.3.2 Dietary phases

Following the initial assessment, athletes adhered to a three-phase dietary approach for weight reduction, followed by a weight recovery phase after the official weigh-in. Key recovery strategies included:

- Liquid consumption: 100 mL/kg, starting immediately after weigh-in.
- Macronutrient distribution: Approximately 70% carbohydrates, 16% protein, and 14% fat.
- Dietary restrictions: Athletes were advised to avoid whole grains and fiber-rich foods.

To monitor compliance, athletes were required to submit daily photos of all meals and drinks consumed via a personalized messaging application (e.g., WhatsApp), and to respond to a short checklist of adherence indicators each evening. Nutritionists reviewed these reports daily and provided corrective feedback in real-time. This method has been adapted from validated remote monitoring protocols used in elite sports nutrition.

Athletes had the autonomy to select their active weight-loss methods, while adherence to dietary and hydration plans was monitored daily via a personal messaging application. Food recall and diet planning were conducted using TBCA® (27) as the primary reference.

#### 2.3.3 Food consumption assessment

The food anamnesis method was employed to assess dietary habits, preferences, allergies, intolerances, bowel and urinary function, prior RWL history, sports engagement duration, training regimen, body composition, and typical versus current weight.

### 2.4 Weight management protocol

**Figure 2:**
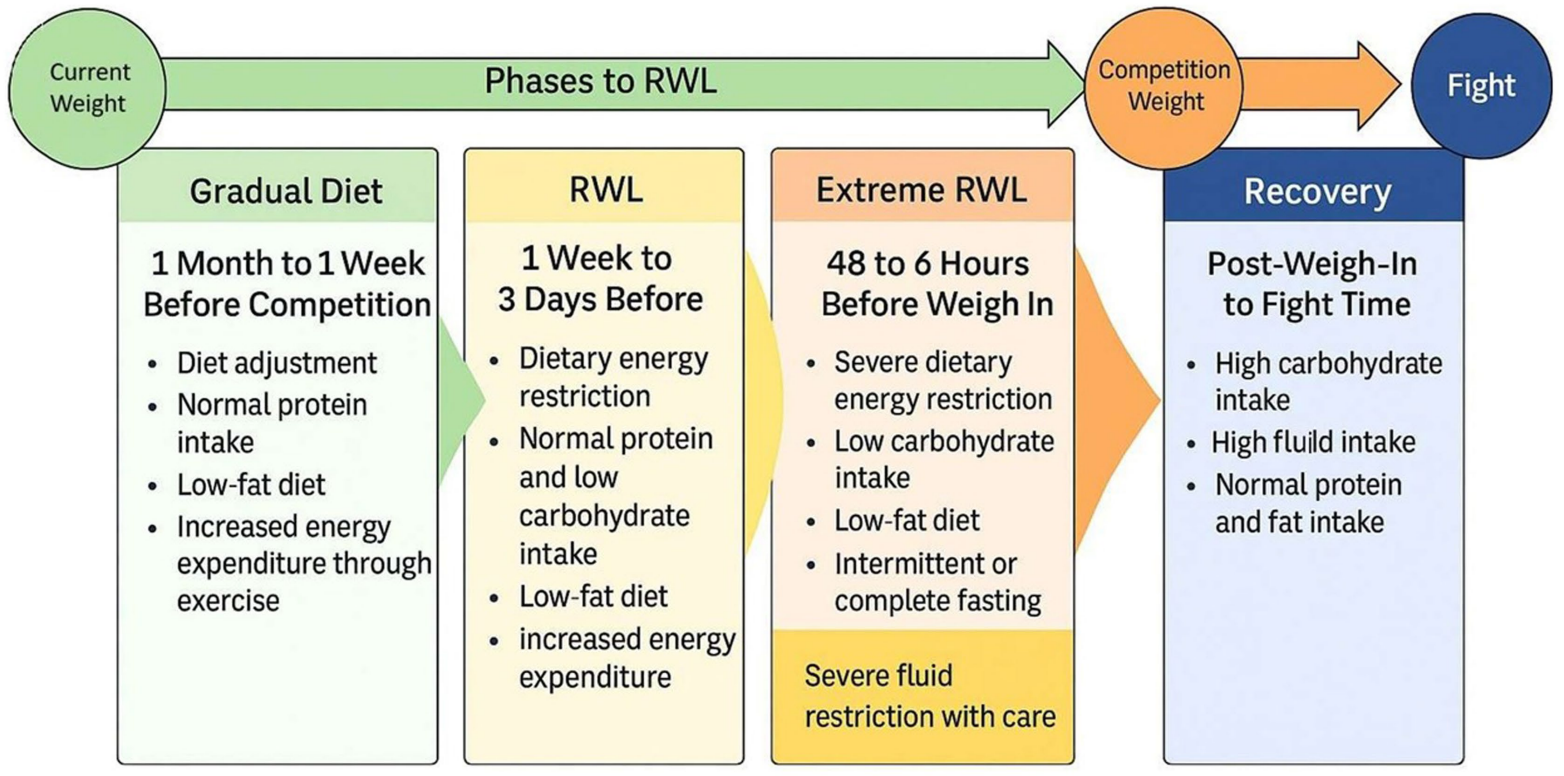
Weight Management Protocol for Rapid Weight Loss in Mixed Martial Arts. RWL, Rapid Weight Loss.

Following the first intervention, the RWL process was categorized into three progressive stages, as outlined in Figure 2, based on the guidelines established by Wright and Garthe (24).

Phase 1:

- Energy reduction: ~30%, equating to 512.7 kcal over an average of 3.4 days.
- Macronutrient adjustments: Decreased consumption of protein, carbohydrates, fats, and fiber.
- Hydration: Initiated “water loading” per Reale et al. (29) guidelines, starting with 7.5 L/day and tapering to 5.5 L/day by phase end.

Phase 2:

- Duration: Average of 2.5 days.
- Energy and macronutrient intake: Further reductions in carbohydrates, protein, fats, and fiber.
- Hydration: Intake decreased from 4.5 L/day to 3.5 L/day.

Phase 3:

- Duration: Approximately 1.9 days.
- Energy and macronutrient intake: Further reductions from Phase 2 in protein, carbohydrates, fats, and fiber.
- Hydration: Started with 1 L/day, tapering to 500 mL/day.

**Figure 3:**
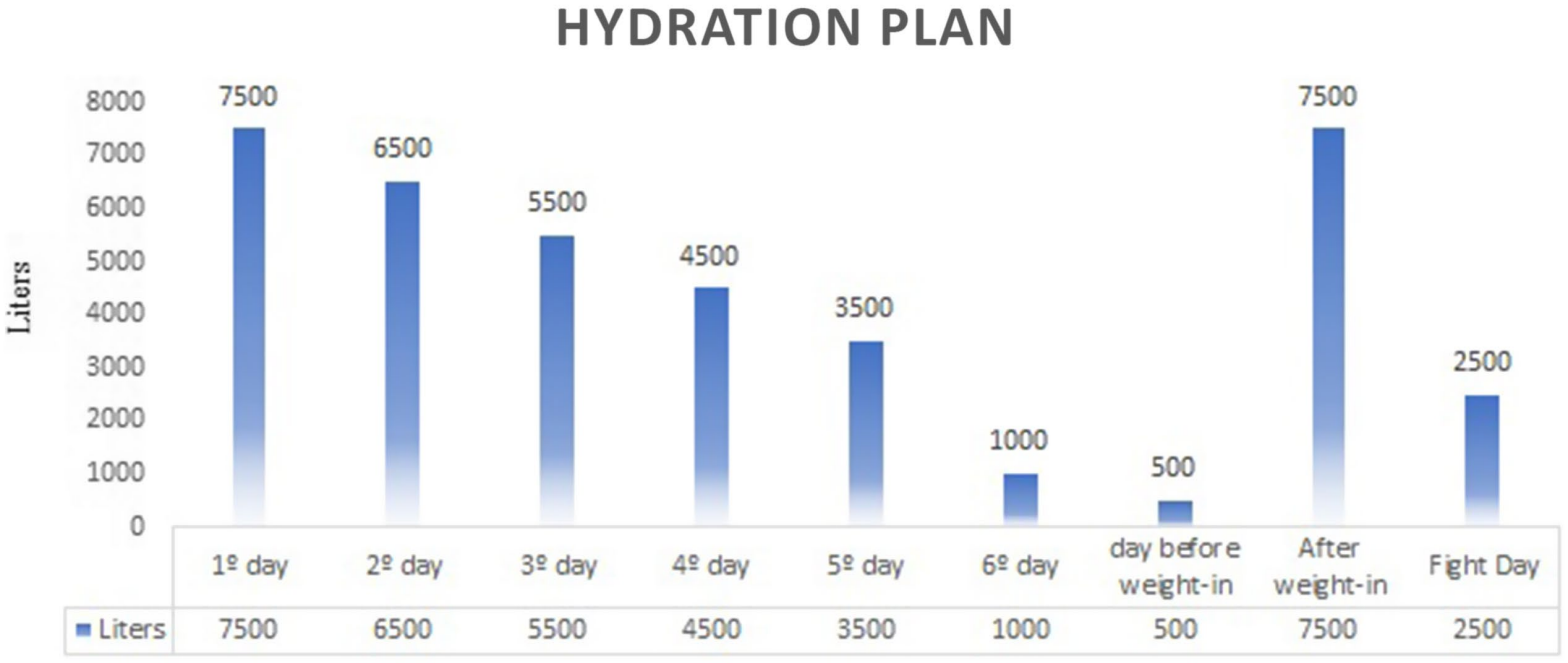
Hidratation plan days and liters a day.

Dietary construction for phases 1, 2, and 3 was individually tailored, reflecting each athlete’s baseline consumption and initial dietary interventions. The average monitoring period spanned 29.7 days, as shown in Table 2, while the hydration plan is illustrated in Figure 3.

### 2.5 Monitoring of recovery phase

Immediately after weigh-in, athletes consumed 1.5 L of electrolyte drinks over 90 min. After this initial rehydration, the solid food phase commenced, emphasizing carbohydrate- and protein-rich foods. Carbohydrates were the predominant macronutrient, followed by protein and fat. Total liquid consumption reached 7 L (±0.5), including electrolyte drinks and fruit juices consumed alongside meals and distributed throughout the day. This recovery plan spanned 24 to 36 h, as detailed in Table 3.

**Table 3:** Distribution recovery phases, with caloric and macronutrient.

Recovery phase			
Variables	Mean	SD	Percentages
Days	1	0.5	
Cal [Kcal]	2146.6	225.1	
Ptn [gm]	87.7	5.5	16
Cho [gm]	379	48.9	70
Lpd [gm]	34.8	2.1	14
Fiber [gm]	39.5	4.4	
Liquid (l)	7.0	0.5	

### 2.6 Statistical analysis

Descriptive statistics included means and standard deviations (SD), with normality assessed via the Kolmogorov–Smirnov test. To analyze differences across multiple time points during the RWL process, a one-way repeated measures ANOVA and Bonferroni *post hoc* test were used. Partial eta squared (*η*^2^) was calculated to determine the magnitude of observed differences, with effect sizes interpreted according to Cohen’s guidelines (small: *η*^2^ = 0.01; medium: *η*^2^ = 0.06; large: *η*^2^ = 0.14) (54).

To verify potential confounding variables, additional between-subjects analyses were conducted. A General Linear Model (GLM) was used to examine the effects of age and sex on body mass across the RWL phases, training volume, and prior weight loss experience. Interaction effects were tested using factorial models to assess whether age moderated the impact of the weight management protocol. Pearson correlation analyses were also conducted to explore associations between age and weight at each phase of the intervention. Correlation coefficients (r) were interpreted as follows: Very weak: 0.00–0.19; Weak: 0.20–0.39; Moderate: 0.40–0.59; Strong: 0.60–0.79; Very strong: 0.80–1.00. For all statistical analysis the Statistical Package for Social Sciences (SPSS, version 20.0) was used, and statistical significance was set at *p* ≤ 0.05 throughout all analyses.

## 3 Results

During the RWL phase, athletes lost an average of 7.25 kg, corresponding to 10.56% of their initial body weight. Following the weigh-in, within the subsequent 30-h recovery period leading up to competition, athletes regained an average of 7.5 kg, equating to an 11.25% increase from their weigh-in weight, as shown in Table 4.

**Table 4:** Descriptive analysis of nutritional variables pre-RWL, post-RWL, post-regain weight.

Weight before RWL	72.38 kg (8.4)*#	-
Height	1.8 mts (0.1)	
BMI before RWL	23.8 w/h^2^ (1.7)	
Kcal before RWL	1812 kcal (501.7)	
Hydration before RWL	7 L (0.5)	
Weight post RWL	64.8 kg (7.9)*#	−10.56% (1.8)
Kcal last day before the official weight	669.5 kcal (167.5)	
Hydration last day before the official weight	750 mL (0.25)	
Post-Regain Weight [24 – 36 h after official weight]	72 kg (8.5)*#	−11.25% (2.9)
Kcal after the official weight	2157.3Kcal (234)	
Hydration after official weight	7 L (0.5)	
Weight Lost [<10 Days]	7.25 kg (1.9)	
Weight Lost [>10 Days]	10.8 kg (2.7)	
Weight Recovered Between Weigh-In and Combat	7.5 kg (1.7)	67.7% V
Diet Time to Combat [Days]	34 days (19.5)	
Age at Which Rapid Weight Loss Began	19.5y (2.5)	

Mean (SD); * = significantly different *p* ≤ 0.05; # = The effect size was calculated by Partial eta squared (Pn2) for all tests.

Adherence to the prescribed hydration and dietary protocols was 100% during both the RWL and weight regain phases. The analysis of variance revealed significant differences in body mass across the three study phases (*F* = 568.761, *p* < 0.001, *η*^2^ = 0.945), indicating a substantial effect size.

Present study verified individual variability in weight loss and recovery responses, standard deviations. Although average weight loss and regain values were similar across the group, the moderate standard deviation (±1.6 kg) suggests some inter-individual variability in response to the protocol. However, no statistical outliers were identified, and the range of responses remained within the expected physiological thresholds. Moreover, the strong correlations between weight measurements at different phases (1 × 2: *r* = 0.989; 1 × 3: *r* = 0.993; *p* < 0.001) indicate a highly consistent pattern of weight change across participants. These findings suggest that although athletes employed a range of RWL techniques, the structured protocol yielded uniform effects in terms of weight manipulation, supporting its reproducibility and standardized application across different individuals.

Within the cohort, athletes employed various RWL techniques, with certain methods being more prevalent than others. The specific techniques and the proportion of athletes employing them during the RWL week are detailed below (Appendix 1):

- Water Loading: The most prevalent method, utilized by 83.9% of the athletes.
- Sauna Use and Gradual Dietary Restriction: Adopted by 74.2% of participants.
- Training Without Plastic Clothing (to induce dehydration): Chosen by 71% of athletes.
- Training with Plastic Clothing: Used by 64.5% of participants to enhance sweating.

Regarding the physiological and physical responses to RWL, athletes reported several symptoms (Appendix 2):

- Fatigue: The most commonly reported symptom, experienced by 58% of athletes.
- Cramps: Reported by 32% of participants during the RWL phase.
- Dizziness: Experienced by 22% of athletes.

Other symptoms, including palpitations, headaches, heartburn, and nausea, were reported by fewer than 15% of participants. Fortunately, no severe symptoms, such as fainting or chest pain, were reported by any athlete during the study.

To assess potential confounding variables that might influence the outcomes of the structured WMP, we conducted supplementary statistical analyses. Specifically, we examined the influence of age and sex on the dependent variables using a between-subjects effects model. The analysis revealed that age had a significant effect on weight variation (*p* < 0.05) across different time points (weight at the beginning of Phase 1, Phase 2, and Phase 3), while sex did not reach statistical significance (*p* > 0.05). Additionally, training volume and prior weight loss experience showed a significant relationship with age (*p* < 0.01), suggesting that older athletes may have adopted more conservative strategies. However, multivariate models did not reveal significant interaction effects between body weight across phases (weight at the beginning of Phase 1 × Phase 2 × Phase 3) and age (p > 0.05), indicating that while age may influence individual weight management strategies, it did not significantly moderate the protocol’s effectiveness. Pearson correlation analyses supported these findings, showing weak to moderate, but non-significant, correlations between age and weight measures (*r* = 0.223–0.257; *p* > 0.1).

## 4 Discussion

Effective weight management, particularly RWL, is pivotal in MMA. The objective extends beyond merely meeting specified weight categories to ensuring athletes achieve peak performance during competition. This study provides critical insights into the weight loss and recovery practices adopted by MMA athletes.

A principal finding was the significant reduction in body weight, averaging a 10.6% decrease from baseline body mass and their recovery of 11.25% from weight-in, decrease weight and recovery well is so important and have a totally relationship with the winner. This observation aligns with findings by Barley et al. (10), who reported that 95% of MMA athletes engaged in RWL practices. The emphasis on weight loss during the final 2 weeks before weigh-in underscores the importance of this period, consistent with the observations of Brandt et al. (6).

Upon evaluating the techniques used for RWL, the study revealed a diverse array of strategies employed by athletes. Water loading was the most common method, consistent with findings by Brandt et al. (6). Additionally, athletes incorporated steam rooms, specific dietary approaches, and targeted training regimens, including training in plastic suits. While these methods align with previous research, their frequency of application varied among participants. Notably, the pronounced dependence on progressive food restriction, as reported by Brandt et al. (6), contrasts with the more diverse strategies observed in the present study. Importantly, the mutual avoidance of pharmacological methods, such as diuretics and laxatives, reflects a shared concern about their potential health risks. Athletes’ experiences during RWL varied; however, fatigue emerged as the most consistently reported adverse effect, highlighting the physical toll and potential performance consequences of improperly managed RWL.

In our study, the post-weigh-in weight regain was equivalent to the athletes’ initial body weight, demonstrating complete restoration following the RWL. Preceding reports indicated that the outcome reflects a structured and effective rehydration and nutritional recovery strategy (20, 26, 30). similarly reported that British MMA athletes lost approximately 8% of their body mass during the pre-competition week, with post-weigh-in recovery averaging 11.7%. Peacock et al. (31) showed the MMA athletes lose on average 5.7% 72 h prior the weight-in, and the recovery was between 7 and 12%, considering different classes, so our recovery looks like on average the others. Furthermore Coswig et al. (26), suggested a positive correlation between weight regain and competitive success in MMA. These findings underscore the importance of structured recovery protocols to mitigate potential performance impairments.

Excessive weight loss, particularly reductions exceeding 5% of body mass, can adversely affect both health and performance, a concern echoed by y Barley et al. (10, 26). While RWL practices remain prevalent in combat sports, non-pharmacological methods, such as sauna use and training in plastic clothing, continue to dominate (32). Most studies identify dehydration as the primary means of achieving RWL (5, 33, 34). theorized that athletes engaging in RWL might exhibit an increased likelihood of success, a hypothesis supported by Koral and Dosseville (35). Interestingly, Maurício et al. (36) reported no significant impact on strength metrics for weight losses ranging from 3 to 5% over short durations.

In the recovery phase following weigh-in, athletes adopted a systematic rehydration and nutritional strategy, prioritizing both fluid intake and carbohydrate-rich foods. Although caloric intake varied from the findings of Matthews et al. (20), the focus on carbohydrate consumption for energy replenishment aligns with broader sports nutrition guidelines. This study, along with findings by Matthews et al. (20) and Barley et al. (10), highlights the efficacy of post-weigh-in recovery strategies, enabling athletes to regain a substantial proportion of their lost weight. Discrepancies observed by y Faro et al. (37) might stem from variations in athlete demographics, suggesting potential differences in recovery patterns based on competitive level.

Establishing a correlation between weight recovery and competitive success further underscores its importance. Both the present study and findings by Brandt et al. (6) and Faro et al. (37), indicate a positive relationship between effective weight recovery and successful performance outcomes Coonor et al. (11), carried out an investigation on the forms and prevalence of rapid weight loss in MMA athletes, considering professionals and amateurs, in which he observed that professionals usually had a weight loss of 8.6% and a regain of 100% of the lost weight, in addition, they observed that the most frequently used methods in weight loss were the gradual diet (62%), and the modulation of liquids such as water loading (62%) and restriction of intake (62%), compared to our method, the participants performed these methods more frequently, as 74.5% performed a gradual diet and 83.9% water loading, while Andreato et al. (38), reported in his observational study with 8 MMA athletes, that they lost an average of 5 kg in the week of the competition and recovered 3.3 kg until the fight, having a greater recovery in the week following the competition, reaching 4.4 kg, with a smaller loss than that seen in this article (7.25 kg) and a smaller recovery, 4.4 kg vs. 7.5 kg, in addition to this comparison, participants reported that the most frequent methods were increased exercise (50%) followed by fluid restriction (37%), both comparative methods differed from the present method due to their smaller loss and different rapid weight loss processes, such as not using the proposed gradual diet methods.

Ribas et al. (30) observed that MMA athletes participating in his questionnaire study reported a rapid loss of 9 kg (11%) of their initial weight, with a 100% recovery after the fight. In comparison with the current method, we can observe that these participants have already reported fluid restriction (72%), followed by sauna (60%), and gradual diet (56%), which were always methods performed by the participants, values still lower than those observed in our study 83.9, 74.2, 74.2%, respectively. Likewise, Santos-junior et al. (39), also through questionnaires, observed 19 Brazilian MMA athletes, concluding that the athletes usually lost 10 kg, 13% of their weight in 20 days before the fight. They did not evaluate weight recovery and had as the most common methods the gradual diet (64.2%), increased training volume (63.1%) and fluid restriction (62.6%), however, in both studies, the weight regained after weighing and before the fight was not verified, which, reiterated by Faro et al. (37), is a factor related to victory. Leaving the sphere of MMA and observing grappling sports such as judo, wrestling, sambo and Brazilian jiu-jitsu, Ranisavijev et al. (40), observed that athletes lose an average of 3.5% of their weight in the week of the competition and that the methods that are always used for rapid weight loss are gradual diet (51%) and increased exercise volume (37%), given this, it seems that both fluid restriction and gradual diet are a routine practice in weight loss phases in MMA and combat athletes, even Zubac et al. (41) proposed the weight loss for 3% in boxers athletes among fluid and food restriction in just 2 days, but the clarity of how it is carried out and even more so, how to recover the lost weight, is something that was not presented in the studies cited, thus increasing the relevance of the present study, which demonstrates a way of carrying out both methods with proven effectiveness in weight loss and recovery compared to other studies.

Beyond performance, recovery plays a crucial role in safeguarding athlete health, as dehydration can compromise cardiovascular and renal function (21, 42). To mitigate these risks, athletes should consume sodium-containing electrolyte beverages to support hydration, particularly following aggressive dehydration processes. Fluid intake should follow the recommended guideline of 1.5 L per kilogram of body weight lost, as outlined by McDermott et al. (43) and Shirreffs and Sawka (44).

In addition to fluid intake, the consumption of nutrient-dense, carbohydrate- and protein-rich foods is essential for replenishing lost minerals and promoting fluid retention. Foods such as fruits, bread, potatoes, meat, eggs, and milk should form the foundation of recovery nutrition, aligning with guidelines established by the American College of Sports Medicine (25, 45).

The complexities of weight management in MMA necessitate a balance between scientific knowledge and strategic planning. Insights from this study and related research emphasize the importance of evidence-based approaches to RWL and recovery. Attaining weight objectives is only one facet of competition readiness; ensuring optimal performance by addressing the physiological and psychological consequences of RWL is equally paramount. This underscores the need for collaboration among athletes, coaches, nutritionists, and other stakeholders to develop safe and effective protocols tailored to individual needs.

It is important to stress that RWL should not be routinely employed, as prolonged low energy availability can lead to relative energy deficiency, impairing bone health, hormonal balance, kidney function, immune resilience, and psychological well-being. These adverse outcomes are well-documented by Cupka and Sedliak (4), Fahrenholtz et al. (18), Kim and Park (46), Lakićević (47), and others. Moreover, RWL can undermine physical fitness, diminish training efficacy, and elevate injury risk (48–50).

To mitigate these risks, RWL should be planned and monitored by a multidisciplinary team, including a nutritionist, physician, coach, and other relevant professionals. As Artioli et al. (13) and Melin et al. (51) emphasize, RWL should be discouraged whenever possible and implemented only under professional supervision.

Several limitations should be acknowledged. The present study used each athlete’s pre-weight-loss body weight as a reference point for post-comparisons across each phase of the weight loss process, rather than employing a separate control group. Although confounding variables did not show statistical significance, factors influencing weight loss, such as training type and frequency, were not controlled but only recorded, as these were determined by each athlete’s coaching team. Second, active weight loss methods, including sauna use, hot baths, and training in plastic suits, were not directly monitored, though athletes commonly reported such practices before nutritional supervision. Although some participants competed in international events, the effectiveness and applicability of the protocol may vary across regions due to differences in hydration practices, access to professional support, and regulatory frameworks (52, 53). The primary intervention focused solely on dietary and hydration aspects, including personalized nutritional prescriptions and continuous communication with both athletes and technical staff.

Although the present study primarily addressed physiological outcomes related to a structured WMP, it did not evaluate psychological factors associated with RWL. For instance, Brandt et al. (6) conducted an exploratory pilot study investigating the relationship between RWL and mood state alterations in professional male MMA athletes. The study compared athletes who engaged in RWL with those who did not, evaluating body weight and mood states (including anger, confusion, depression, fatigue, tension, and vigor) across four time points: 30 days before competition, at the official weigh-in, 10 min pre-fight, and 10 min post-fight. The findings indicated that athletes undergoing RWL experienced consistently higher levels of confusion and total mood disturbance throughout all stages (6). Despite these psychological disturbances, RWL did not appear to impair athletic performance. The study provides important evidence that RWL practices, while not necessarily affecting competitive outcomes, are associated with potentially dysfunctional mood states, reinforcing the need for protocols that consider not only physical but also psychological health (6). Future studies should incorporate validated psychological instruments to evaluate emotional responses, stress levels, and cognitive functioning throughout the weight management process.

While this study was conducted with professional MMA athletes based in Brazil, cultural and dietary factors may influence the generalizability of the findings to broader international populations. Brazilian athletes may follow distinct nutritional habits, food availability, and cultural attitudes toward weight cutting, which could affect both adherence and response to structured protocols. Although some participants competed in international events, the protocol’s effectiveness and applicability may vary in different regions due to differences in cuisine, hydration practices, access to professional support, and regulatory norms. Therefore, future studies should aim to replicate and adapt this structured WMP in diverse cultural contexts to evaluate its global applicability and effectiveness.

As a practical application, although weight-cutting ideally should not be used in combat sports, it is a common and culturally ingrained practice in these disciplines. Therefore, we do not recommend applying these methods to children or adolescents under 18 years of age, or to athletes with existing medical conditions, such as kidney issues. In this study, we presented the effects of a RWL protocol limited to no more than 10% of the athlete’s initial body weight, with a required recovery period of at least 24 h. The process should follow a structured progression: first reducing calories, then carbohydrates, and finally hydration. Above all, it is crucial to implement this protocol under professional supervision to minimize potential health risks. In our study, athletes were monitored accordingly to assess both the weight loss process and any associated symptoms or side effects. Therefore, we emphasize that the practical application of this protocol should involve weekly monitoring by a qualified nutritionist and physician.

## 5 Conclusion

Our study provides evidence that the described method is not only effective for RWL among combat athletes but also facilitates complete weight recovery by professional MMA athletes. Furthermore, it is particularly noteworthy that this weight loss and subsequent recovery did not negatively affect competition outcomes, underscoring the strategic effectiveness of the method.

Present findings highlight the efficacy of the proposed weight management approach, positioning it as a viable and efficient model for athletes aiming to optimize weight control while maintaining competitive readiness. To prioritize athlete well-being, we recommend that MMA and other combat sports athletes avoid engaging in RWL whenever possible. This guideline would not only optimize strength and power performance but also minimize potential health risks, ensuring that athletes maintain peak physical condition throughout their weight management.

As a suggestion for future research, studies should incorporate a longitudinal design to assess the effects of weight loss and recovery on both physiological and psychological parameters, such as hormonal balance, cognitive performance, mood states, and the well-being of athletes.

## Data Availability

The datasets presented in this study can be found in online repositories. The names of the repository/repositories and accession number(s) can be found in the article/supplementary material.
