# Supplementary material for: An alternative structured weight management protocol to rapid weight loss in mixed martial arts: a prospective interventional study of pre-competition weight management strategies in professional athletes
Source: Front Nutr. 2025 Oct 8;12:1581698. doi: 10.3389/fnut.2025.1581698 (PMC12540128; doi:10.3389/fnut.2025.1581698)
Supplement: Supplementary file 2 [file Table_1.docx]

Appendices

Appendix 1

The specific techniques and the proportion of athletes employing them during the RWL week are detailed below

|  | Yes | No |
| --- | --- | --- |
| Gradual diet | 23 (74.2%) | 8 (25.8%) |
| Restritctive diet | 9 (29%) | 22 (71%) |
| Fasting | 8(25.8%) | 23 (74.2%) |
| Jump meals | 1 (3.2%) | 30 (96.8%) |
| Fluid restriction | 11 (35.5%) | 20 (64.5%) |
| Training with plastic suits | 11 (35.5%) | 20 (64.5%) |
| Training to dehydration | 22 (71%) | 9 (29%) |
| Water loading | 26 (83.9%) | 5 (16.1%) |
| Steam room | 23 (74.2%) | 8 (25.8%) |
| hot bath tub | 11 (35.5%) | 20 (64.5%) |
| Laxatives | 0 (0%) | 31 (100%) |
| Diuretics | 2 (6,5%) | 29 (93,5%) |
| termogenics | 7 (22,6%) | 24 (77,4%) |

Appendix 2

Regarding the physiological and physical responses to RWL, athletes reported several symptoms

|  | Yes | No |
| --- | --- | --- |
| Dizziness | 7 (22.6%) | 24 (77.4%) |
| Faiting | 0 (0%) | 31 (100%) |
| Fatigue | 18 (58.1%) | 13 (41.9%) |
| cramps | 10 (32.3%) | 21 (67.7%) |
| palpitations | 5 (16.1%) | 26 (83.9%) |
| chest pain | 0 (0%) | 31 (100%) |
| headache | 4 (12.9%) | 27 (87.1%) |
| stomachache | 1 (3.2%) | 30 (96.8%) |
| nausea | 3 (9.7%) | 28 (90.3%) |
